# Validity and reliability of the (adjusted) Impact on Participation and Autonomy questionnaire for social-support populations

**DOI:** 10.1186/s12955-019-1106-0

**Published:** 2019-02-26

**Authors:** Lucienne Berenschot, Yolanda Grift

**Affiliations:** 1 Berenschot Onderzoek en Advies, Hogekampweg 50, Apeldoorn, 7316MT the Netherlands; 2 Faculty of Law, Economics and Governance, University Utrecht School of Economics, PO Box 80125, Utrecht, 3508 TC the Netherlands

**Keywords:** Social support, Participation, Autonomy, IPA, Impairment, Validation, Factor analysis

## Abstract

**Background:**

Decentralisation of social support and budget cuts spurred interest in outcome-oriented payment systems in the Netherlands. Hence, measurement of relevant outcomes, such as self-reliance and participation, is needed. The Impact on Participation and Autonomy (IPA) questionnaire for rehabilitation clients was adapted for social support, called IPA-MO, and its validity and reliability were tested among social support clients in eight municipalities in 2014 and 2016.

**Methods:**

The total research sample comprised of 4120 persons. Homogeneous subgroups were distinguished based on 1) disability and 2) age. Exploratory factor analysis (PCF) was used to identify domains for IPA-MO. Model fit was assessed with Confirmatory Factor Analysis (CFA) using structural equation modelling.

**Results:**

PCF revealed that the IPA-MO model consists of all five original IPA domains: Autonomy indoors, Family role, Autonomy outdoors, Social life and relations, and Work and education. As a result of new items added, a new, sixth domain was developed: Financial autonomy. Due to high non-response on Work & education, construct validity was first tested for a five-domain IPA-MO model. The composition of the IPA-MO domains showed slight differences: the item on ‘mobility indoor’ shifted from Autonomy indoors (IPA) to Family Role in IPA-MO. The item on reciprocity shifted from Social Life and relations (IPA) to Autonomy outdoors (IPA-MO). Internal reliability was confirmed for all domains (Cronbach’s alpha >.80). CFA showed acceptable construct validity of the five-domain IPA-MO model for the social support population (CFI .936, TLI .925, SRMR .051), all age groups and most disability-based groups. Construct validity including Work & education was tested for 234 participants. Then, PCF revealed six domains and the model fit was acceptable (CFI .915, TLI .903, SRMR .067).

**Conclusions:**

IPA-MO is a valid and reliable instrument to assess outcomes of social support. Further research on the domain Financial autonomy is needed. Social-support clients are numerous and dispersed and, in spite of the best intentions, hard to involve widely in policy processes. A valid outcome measure offers municipalities possibilities to gain insight in social costs and benefits of new policies. Outcome measurement also allows to contract bundled-services of providers, thereby changing the incentives for providers from increasing production to much needed innovation. Taking the perceptions of autonomy and participation of social-support clients as the acid test for the effectiveness of social support policies, may prove a serious game-changer in politics.

## Introduction

Under the Dutch Social Support Act (“Wet maatschappelijke ondersteuning – Wmo”), public social-support services have progressively been transferred to local governments. Since 2006, municipalities manage a wide range of non-medical facilities to support adult inhabitants living with a disability in the field of domestic tasks, mobility and social life. In 2015, facilities for individual and group guidance also became part of Wmo [1]. Simultaneously, the Participation Act and Youth Act enhanced the municipalities’ mandate in the field of employment and youth care [2, 3].

The decentralisation of responsibilities in the ‘social domain’ is related to national policies that aim to reform the welfare state, whereby people living with disabilities should participate in ‘normal life’ and be as self-reliant as possible. Government policies should foster an enabling environment, and their support should strengthen people’s ability to participate. This is a major change from the past, when government policy was oriented towards taking over the care for these people, based on the assumption that inactivity is a logical consequence of impairments [4]. This change in national policies is in line with the paradigm change introduced by the WHO in 2001 with the ratification of the International Classification of Functioning, Disability and Health (ICF) [5]. The new emphasis on social functioning of people living with a disability is expected to increase their quality of life and contain the rise in public care costs [1].

Along with the decentralisation of social support, budget cuts were imposed as municipalities were expected to organise services in a more efficient way. This resulted in a renewed interest in integrated service delivery and outcome-based payment (instead of the current production-related reimbursement systems) [6]. In a study commissioned by the Dutch Ministry of Health, the possibilities for outcome-driven social-support systems were explored [7]. Based on Brickley et al. [8] three key elements for outcome-driven systems were identified: adequate organisational design, well-aligned incentives in payment models and reliable assessment of outcomes. The Impact on Participation and Autonomy (IPA) questionnaire was identified as a promising instrument to assess relevant outcomes [7]. Originally IPA was developed in the Netherlands in order to identify interventions with rehabilitation clients from their own perspective [9]. Rehabilitation science and practice have a long-standing tradition in enhancing the participation in society of people with disabling conditions, which is also the newly adopted focus in social support. The body of knowledge and experience developed in rehabilitation science may therefore be useful in developing new social-support strategies oriented towards self-reliance and participation.

Specific reasons for selecting the IPA instrument in this study were fourfold:

1. IPA offers a broad overarching scope that matches target groups and responsibilities of municipalities under the Social Support and Participation Acts: it is a generic instrument (designed for adults with any disabling condition) and covers all relevant life domains (domestic tasks, mobility, social life and relationships, self-care, income, work, education and leisure);
2. IPA is a validated questionnaire that assesses the self-perceived degree of participation of individuals. A distinctive quality is the inclusion of autonomy - the extent to which an individual has control over the way he lives – as inseparably linked to participation [9]. Autonomy is closely linked to the self-reliance that the new social-support policies in the Netherlands seek to reinforce [10];
3. IPA measures participation in terms of ‘performance’, described as ‘what an individual does in his or her current environment’. The current environment is understood to include assistive devices, personal assistance and public facilities used by the individual [5]. In other words, performance reflects how people function in everyday life *with* the available support. As such, it may serve as an indicator of the effectiveness of social support policies;
4. IPA is a self-reported questionnaire. Subjective measures are increasingly seen as preferable to assess ‘needs met’ for qualitative goals such as quality of life [11] and participation [12]. Whiteneck [13] even argues that participation, by its very nature, can only be evaluated by self-report. The Social Support Act (Wmo 2015) itself also stresses the importance of clients’ perception in quality assessment of social support [10].

The objective of this study is to assess the reliability and validity of the IPA instrument to measure participation of social-support clients on a population level.

The remainder of the article is set up as follows. In the Methods section first, the original IPA questionnaire and its validation for rehabilitation clients are presented. Second, the adaptations made in the questionnaire for use among social-support clients are discussed. The third section covers a description of the data collection and the factor analysis used. In the Results section, the analyses are presented. The article ends with conclusions and a discussion of our findings.

## Methods

Between August 2014 and October 2016, eight municipalities wanted baseline information before the Social Support 2015 and Participation Act came into force. Using the IPA questionnaire surveys were implemented among large cohorts of social support clients with physical, mental and intellectual disabilities as well as persons who are unable to earn a living and depend on income support. All participants live in the community (not in institutional homes).

### The Impact on Participation and Autonomy instrument: the original questionnaire

The Impact on Participation and Autonomy questionnaire assesses two different aspects of participation: perceived participation and perceived problem-experience [14]. Perceived problem-experience is scored independently and the results are used to establish goals in individual rehabilitation programmes that reflect personal preferences. In this article the focus is on perceived participation.

Perceived participation is assessed by 32 items, grouped together in 9 sections: mobility (4; 1a-1d), self-care (5; 6a-6e), domestic tasks and role (6, 2a-2f), income (1; 3d), leisure (1; 4), social contacts and relations (7; 5a-5f,5 h), helping others (1; 5 g), work (5; 7a-7e), education (1; 8) and a final item on overall autonomy and participation as perceived by the participant (1; 9a). The items are phrased in a way that emphasises control over tasks and activities (decisional autonomy) rather than whether they can be implemented with or without support (executional autonomy). As an illustration, an item on personal care is (6a or 6b): “my chances to decide when I get washed and dressed are …” Items are scored on a five-point Likert scale, ranging from very good, good, fair, poor to very poor.

Cardol [14] found that 26 of the 32 perceived-participation items load onto four domains called ‘participation domains’: Autonomy indoors (AI), Family role (FR), Autonomy outdoors (AO), and Social life and relations (SOC). She assumed a fifth participation domain, Work and education (WORK) but could not confirm it since few participants in her study had employment. The five latent domains contribute to the overarching concept of participation. Cardol et al. [9] validated the IPA questionnaire extensively. Psychometric properties such as internal reliability and test-retest reliability proved to be good on a domain level, though some items were psychometrically weak. Responsiveness to change, one of the aspects Cardol addressed, was good for three domains (WORK, AO, FR) while moderate to no responsiveness was found for two domains (AI, SOC). Convergent and divergent validity were tested with instruments such as London Handicap Scale, Sickness Impact Profile and Short Form-36 and were generally confirmed [14].

The IPA questionnaire has since been validated and adopted in many countries both in and outside Europe [15–22]. Most studies focused on rehabilitation clients with well-defined, specific physical impairments. Construct validity was tested with various methods: exploratory factor analysis, the Rasch methodology, Principal Component Analysis and Confirmatory Factor Analysis. Only the UK study [15] validated the Work and education domain. Most studies confirm the model of Cardol, with the exception of an Iranian study that found participation domains were clustered into two dimensions: performance-based and social-based participation [20]. Wilkie et al. [23] conclude that the instrument has good face validity and its measurement of participation is comprehensive and relevant according to patients. He recommends further testing of construct validity and responsiveness since study populations have not been very large and Confirmatory Factor Analysis has hardly been used in validity studies.

### Adapting IPA for use in social-support populations

Feasibility of the IPA questionnaire for social-support clients was tested in a pilot among some 500 participants in 2013. This resulted in the following adaptations to the questionnaire:

- the section on self-care was positioned more towards the end of the questionnaire, in order to avoid participants feeling addressed as ‘patients’;
- three new items were added in the section on income (3a-3c) at the request of municipalities, who are responsible for debt prevention and relief. The new items were derived from a validated instrument for assessment of financial capacities of individuals developed by Mesis [24];
- the item on intimate relationships (5f) was rephrased into ‘my chances to give and receive love and affection’, thus avoiding the impression that the item refers to sexual relationships only and broadening the scope to all affective bonds;
- the items on perceived problem-experience were left out;
- an extensive inventory of support available to participants (informal care, assistive devices, public facilities, privately acquired services and personal assistance) was included.

The adapted IPA questionnaire contains 35 items on perceived participation and is used in the data collection among social-support populations.

### Data and the process of validation

This section starts with defining the social-support population by disability type. Next, the data collection, the assessment of clients by disability type and a description of the data are presented. In the last part we describe the process of validating the IPA questionnaire and the methods used.

#### Defining the social-support population

The survey population comprised of people who receive individual or collective guidance (the ‘new’ client groups for municipalities under the Wmo 2015), as well as persons with mild physical disabilities (‘traditional’ Wmo clients). Also, people who have received income support for a long period (> 1 year) were included based on the assumption that their participation might be hampered due to physical or mental problems [25].

Wmo-clients were divided into four groups: people with mild physical disabilities – mainly elderly – used ‘light’ municipal support such as assistive devices, special transport facilities or domestic help. Participants with severe physical disabilities suffered from diminished physical or mental vitality as a result of severe motoric handicaps, dementia, chronic and progressive illness (e.g. rheumatism, ALS) or acquired brain injury. They received more intensive support such as individual and/or collective guidance, in order to enable them to live in the community. The ‘mild intellectual disabilities’ group included people who were socially vulnerable as a result of restricted intellectual capacities. Participants with mental-health problems dealt with addictions, psychotic disorders, anxiety attacks or behavioural problems such as autism or ADHD [26]. The latter two groups received individual guidance and guided group work so as to support their daily functioning. Participants recruited among the Participation Act clients form a fifth group.

#### Data collection

Between August 2014 and October 2016, surveys with the adapted IPA questionnaire were implemented among large cohorts of social support clients in eight municipalities that wanted baseline information before the Social Support Act 2015 and the Participation Act came into force. Three of them implemented a follow-up survey after 2 years. Municipalities belong to population categories 20,000–50,000 (6), 100,000–150,000 (1) and 150,000–200,000 (1) inhabitants. Four municipalities are mainly urban and four have a rural character.

Participants from categories without readily-available registration (guidance clients in 2014/15, clients with mild physical disabilities in all years) were recruited through care providers. For income-support clients and guidance clients in the follow up surveys, participants were recruited by random samples. Municipalities approached all income-support clients who had received this support for 1 year or longer. For guidance clients systematic samples were drawn from municipal registrations in 2016. All participants received a questionnaire (hard copy) accompanied by a letter of invitation signed by the alderman of their municipality. The majority of questionnaires were self-administered. People with (mild) intellectual disabilities got support from family members or care providers, who received instructions stressing that client perspective should prevail. Though interference of the helpers’ view is a risk of this procedure, we considered it worthwhile to try and capture the perspective of this group too.

All data from the baseline and follow-up surveys (*n* = 4660) were combined in a single database. Records with 4 or more sections with invalid response^1^ (*n* = 75) as well as those of respondents under the age of 18 (*n* = 9) were removed. Of the remaining 4576 records, income-support clients of one baseline survey (*n* = 341) were excluded since this group was not limited to long-term income-support. Wmo-respondents that did not report use of formal support (*n* = 110) or whose type of disability could not be assessed (*n* = 5) were also removed.

#### Assessing disability type

In 2014/2015, information on the type of disability of participants was deduced from the specialisation of their provider organisation. Participants in the follow-up surveys in 2016 were scored on disability based on available data (support provided, provider organisation, age, personal remarks). Each respondent was included in one disability category. However, it should be noted that these categories are not completely mutually exclusive. People living with severe physical disabilities may also have mental problems (such as depression), income-support clients sometimes have intellectual disabilities, people living with ADHD or autistic disorders are sometimes treated as having intellectual disabilities and on other occasions as having mental problems.

In Table 1 the distribution of the research sample by regional characteristics and by disability type or income-support is shown.

**Table 1:** Origin of the respondents by regional characteristics and by disability type or income-support

Year	Type of survey	Region of country	Characteristics of the municipalities		Number of respondents according to disability type				
			Population class (*1000)	Urban/ rural	Mild physical	Severe physical	Cognitive	Mental	Income-support
2014	Baseline	West	100–150	Urban	330	118	40	64	230
			20–50	Rural	24	20	10	19	34
		East	150–200	Urban		187	312	215	
		North	20–50	Rural	213	51	24	12	
			20–50	Rural	210	52	37	10	
2015	Baseline	West	20–50	Urban		50	41	31	
			20–50	Urban		47	32	43	
			20–50	Rural		29	18	19	
2016	Follow up	East	150–200	Urban	41	154	210	190	189
		West	100–150	Urban	309	87	30	24	233
			20–50	Rural	76	21	< 10	< 10	27
Total		3 regions	8 municipalities		1203	816	757	631	713

Source: IPA-MO database, 2014–2016

Given the size and variety in geographical origin of the samples, we assume that the subgroups in our study population are representative for the respective disability or age category. Our total study population, however, is not representative for either the national level nor any of the local communities where the surveys were held. Compared to the national level, elderly Wmo-clients and the group on income support are underrepresented (Table 2). At the municipal level, composition of social-support populations varies substantially due to local factors such as labour market, age distribution and presence of care institutions [27].

**Table 2:** National social-support clients (2015) and research population [38, 39]

	Sub group	National population		Research population	
Social Support Act	20–64 years	201,000	21%	1736	42%
	65–80 years	250,000	26%	768	19%
	80+ years	235,000	24%	834	21%
	Age unknown			69	2%
Participation Act	> 1 year	288,720	30%	713	17%
Total		974,720	100%	4120	100%

Source: IPA-MO database, 2014–2016 and CBS [27])

### Creating subgroups: by disability type and by age

The study population is highly heterogeneous, as it covers a variety of disabilities. Moreover, it covers a wide age spectrum (18–103 yrs) whereas Cardol [14] restricted participation in her sample to the age of 18–75. We chose to include (very) old people since they form an important segment of the population that receive assistance under the Social Support Act^2^ Besides, the validity and relevance of the IPA questionnaire for elderly people (up to 99 yrs) is confirmed by Ottenval Hammar [28].

In order to analyse more homogeneous groups, two approaches were taken. The first was to distinguish participants by the type of disability, as described above. A second approach distinguished participants by age. The life cycle theory suggests that people face different needs and challenges in subsequent stages of their lives [29]. As a consequence, intensity and domains of participation vary in different phases of life [30]. Additional pragmatic advantages are objectivity and mutual exclusiveness of age categories, and the fact that no information on the type of disability is needed. We distinguished five age-groups: young adults (18–35), 2 groups for grown adults (36–50 and 51–66) and 2 groups for late adults (67–80 and 81+).

People with mild physical disabilities are the most numerous group; in terms of age, the group aged 51–66 years is largest. Mental problems and intellectual disability prevail in younger age groups whereas in older groups, physical disabilities are predominant (Table 3).

**Table 3:** Cross tabulation disabilities and age groups in research population

Age (years)	Disability type				Income support	Total
	Mild physical	Severe physical	Cognitive	Mental		
18–35	29	28	271	146	78	552
36–50	52	53	252	228	254	839
51–66	147	150	179	201	371	1048
67–80	417	273	40	38	0	768
81 +	538	291	2	3	0	834
Missing	20	21	13	15	10	79
Total	1203	816	757	631	713	4120

Source: IPA-MO database, 2014–2016

Some characteristics of the data are given in Table 4. Elderly people often use a range of state and private services to facilitate their daily functioning. Many of them also get informal help from friends and family on a regular basis. For the younger groups – many of them with cognitive or mental impairments -, guidance is the main form of state support. Informal help is most common among the 18–35 year-olds and decreases among older adults (36–66 years). Labour participation for participants in the working age is low: 15–23% have a salaried job, 30–40% of participants have unpaid activities, either in guided workshops or voluntary work. Almost half the participants (49%) under the age of 67 have no working activities at all.

**Table 4:** Descriptive statistics of the social-support IPA-MO database

	All	Mild physical disability	Severe physical	Mild intellectual	Mental impairment	Long-term income support	18–35 yrs	36–50 yrs	51–66 yrs	67–80 yrs	81+ yrs
n=	4120	1203	816	757	631	713	552	839	1048	768	834
Gender											
Female	59%	70%	61%	49%	52%	56%	50%	55%	54%	64%	72%
Male	41%	30%	39%	51%	48%	44%	50%	45%	46%	36%	28%
Age											
Mean (yrs)	59,8	75,6	72,3	42,0	45,9	50,4	27,5	43,7	58,2	74,3	86,2
St.dev	20,0	14,0	15,9	14,6	13,4	10,5	4,9	4,2	4,4	4,0	4,1
Range	18–103	18–103	18–102	18–92	18–85	20–65	18–35	36–50	51–66	67–80	81–103
Living											
Alone	54%	58%	48%	44%	64%	59%	37%	47%	63%	50%	65%
With partner	25%	33%	41%	18%	12%	10%	13%	15%	21%	44%	30%
With family	19%	8%	9%	35%	21%	31%	46%	34%	14%	6%	5%
Other	2%	1%	2%	4%	3%	1%	4%	4%	2%	–	–
Work											
None	65%	87%	84%	35%	50%	56%	40%	43%	59%	87%	97%
Paid job	12%	5%	3%	25%	15%	15%	17%	23%	16%	1%	–
Guided work (unpaid)	13%	1%	6%	34%	22%	8%	34%	21%	14%	4%	–
Voluntary activities	10%	7%	6%	5%	13%	21%	9%	12%	15%	8%	2%
Caregiving tasks											
Yes, on regular basis	11%	7%	5%	13%	14%	19%	14%	17%	14%	6%	3%
Sources of income^a^											
Retirement pension	58%	84%	73%	6%	9%	2%	–	–	9%	97%	99%
Salary	11%	6%	5%	24%	15%	12%	15%	22%	15%	2%	2%
Incapacity allowance	27%	10%	18%	64%	49%	6%	63%	41%	35%	–	–
Income support	27%	5%	6%	13%	32%	94%	27%	47%	49%	2%	–
Using support:											
Assistive devices	41%	69%	70%	16%	14%	10%	7%	14%	32%	64%	80%
Informal help^b^	35%	37%	51%	41%	27%	15%	40%	27%	24%	38%	52%
Private services	26%	42%	47%	14%	10%	5%	7%	8%	15%	38%	60%
Special transport	36%	48%	60%	33%	18%	8%	15%	18%	32%	53%	57%
Domestic help (subsid)	44%	75%	65%	23%	27%	8%	7%	20%	37%	66%	83%
Personal assist.	63%	29%	91%	96%	93%	26%	84%	70%	58%	55%	54%

Source: IPA-MO database, 2014–2016

^a^more than one source possible

^b^on a regular basis: at least once a week

#### The validation process

In order to validate the adapted IPA questionnaire we proceeded as follows.

First, we tested domain reliability and validity of Cardol’s model [14] with the original IPA items.

1. validation of the original IPA for 4 domains: Autonomy indoors, Family role, Autonomy outdoors and Social life and relations (internal reliability, factor structure and loadings and goodness of fit) Validation is based on the entire research sample;
2. validation of the original IPA for 5 domains, including Work and education (internal reliability, factor structure and loadings, goodness of fit). This analysis is based on the (small) group of participants that responded to all items on Work and education (*n* = 234);

Next, the adjusted IPA questionnaire with the new items on income (3a-c) was tested. We expected the new items to form a new domain together with the original item on income (3d). We excluded the final item “my possibilities to live the life I want” (item 9) from the model as we consider it to be distinct in nature: as a concluding item, it is meant and phrased as an overall appreciation rather than a predictor of perceived participation. Reliability of the IPA domains is analysed and Principal Component Factoring (PCF) is used to explore the new model structure. Results are tested on goodness of fit by applying CFA using structural equation modelling (Sem).

3) validation of the adapted IPA model including the new items on income and excluding the final item: exploratory analysis of factor structure and loadings, internal reliability of adapted/new domains, goodness of fit. This analysis is done for the whole research population, excluding the items on work and education;
4) validation of the adapted IPA model including the Work and education domain, the new items on income and excluding the final item: exploratory analysis of factor structure and loadings, internal reliability, goodness of fit. This analysis was done for the same participants as in 2).

Lastly, based on the resulting version of the IPA instrument, factors are constructed for its domains: for the whole research sample, the disability groups as well as for the five age categories.

Construct validity was analysed by Principal Component Factoring (PCF) and by Comparative Factor Analysis fitting a structural equation model (CFA by Sem). Goodness of fit was assessed with the usual statistics such as the Comparative Fit Index (CFI), the Tucker-Lewis Index (TLI) and the standardised root mean squared residual (SRMR). In line with Hu and Bentler [31], SRMR< .08 in combination with CFI or TLI > = .95 is used as a cut-off criteria for a good model fit, SRMR <.08 in combination with CFI/TLI > = .90 for acceptable fit.

Stata 15 was used for all analyses.

## Results

In this section we present the results of our analysis as follows. First we validate the original IPA with 4 domains (excluding Work and education) and 5 domains (including Work and education) based on respectively 26 and 32 items of the original IPA questionnaire. For the 4-domain model the whole research sample is used. For the 5-domain model the analysis is based on the (small) group of participants that responded to all items on Work and education (*N* = 234).

Next we validate the adapted IPA model including the new items on financial issues and excluding the final item (see Appendix). Using explorative factor analysis, as expected a new domain emerged - Financial autonomy. Construct validity of a model with and without the Work and Education domain was tested using the whole research sample. Having investigated and shown the validity of the adapted IPA model this section concludes with a short description of the predicted domain scores for the whole research sample, and the subgroups based on disability and on age.

### Reliability and validity of the original IPA model for social-support population

#### 4 domain IPA model: autonomy indoors, family role, autonomy outdoors, and social life and relations

Explorative factor analysis using PCF was used to test validity of the 4-domain IPA model. Following Cardol, we used orthogonal rotation. In Table 5 the results are presented. Results by and large sustained the IPA model. Variance explained is 66%, comparable to that found (67%) by Cardol [14]. Misfits occurred with both items on mobility indoors of the AI domain and the item on financial independence in the FR domain. The latter was found psychometrically weak in Cardol’s study as well [14]. The remaining 23 items showed the highest rotated loadings (ranging from .44 to .85.) on the expected domain. Five of them had high loadings (> = .40) on other domains as well.

**Table 5:**
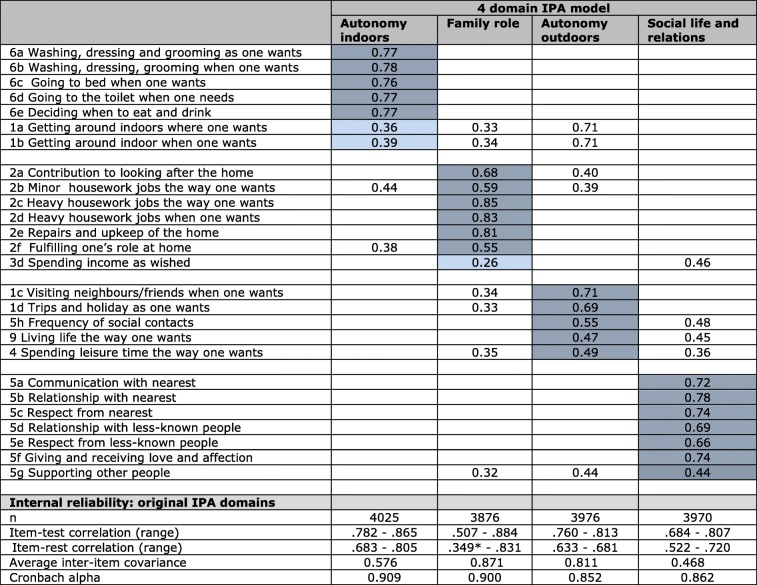
Rotated factor loadings original IPA model for social-support clients. Principal component factoring, Varimax rotation *n* = 3301

items not (optimally) corresponding to a specific domain as found by Cardol [14]

coinciding items for social-support sample

Source: IPA-MO database, 2014–2016

^a^only factor loadings > .32 are shown in non-expected factors (0.32 is suggested as minimum loading of an item in factor analysis [40])

The four IPA domains showed good internal reliability, with Cronbach’s alpha between .85 and .91. Item-test correlations ranged from .51 to .87. Item-rest correlations was low (<.50) for item 3d “spending money as wished” in FR. Average inter-item covariance was high (> 50) in AI and very high (>.8) in FR and AO (Table 5). This was an indication that items were very similar to each other and possibly redundant [32].

CFA showed poor fit indices for the 4-domain IPA model (CFI .791, TLI .768, SRMR .091). CFA for individual domains revealed poor indices for AI and SOC. High error covariances between pairs of items were found in all domains: 1a-1b and 6a-6b in AI, 2c-2d in FR, 1c-1d and 4 and 9 in AO, 5a-5b, 5b-5c and 5d-5e in SOC. In five pairs of items on an identical subject and sometimes even phrased using the same words,^3^ one of the items was eliminated (1a, 2c, 5b, 5e, 6b). The items 1c-1d (visits and trips) and 4 and 9 (leisure time and overall quality of life) were considered to cover distinct concepts and therefore maintained. Instead, we opted for adjusting the error covariance between item 1c-1d. These modifications resulted in good fit for all domains (Table 6; first panel).

**Table 6:** Goodness of fit, including the comparison of domains based on the original (26-items), and adapted (21-items) IPA questionnaire

	4-domain IPA							
	Autonomy indoors		Family role		Autonomy outdoors		Social life and relations	
	original	ex 1a,6b	original	ex 2c	original	covariance 1c & 1d	original	ex 5b, 5e
# items	7	5	7	6	5	5	7	5
n=	3922	3938	3711	3718	3715	3715	3846	3881
CFI	.711	.987	.953	.973	.941	.985	.842	.961
TLI	.567	.973	.930	.955	.881	.963	.764	.923
SRMR	.110	.021	.036	.026	.044	.022	.079	.034
Item-test correlation	.78–.87	.75–.85	.51–.88	.54–.86	.76–.81		.68–.81	.71–.81
Item-rest correlation	.68–81	.57–.77	.35–.83	.36–.78	.63–.68		.52–.72	.53–.68
Average inter-item covariance	0.59	0.52	0.87	0.80	0.81		0.47	0.48
Cronbach’s alpha	0.91	0.87	0.9	0.876	0.852		0.862	0.808
Mean score	3.83	3.86	3.14	3.19	3.05		3.5	3.45
SD	0.79	0.78	0.98	0.96	0.98		0.74	0.77
Pairwise correlation between domains	0.99		0.99		1.00		0.98	

Source: IPA-MO database, 2014–2016

The modified domains were tested on internal reliability. Cronbach’s alpha is good (> .80) for all domains, though slightly lower than in the original domain. Item-rest correlation remained low for the financial item (3d, FR), average inter-item covariance was still high for FR (.80) (Table 6, second panel). As mean scores of the short-version and original domains did not differ much and were highly correlated (.98–1.00; Table 6, third panel), we conclude that the short version is a reliable and conceptually similar alternative for the original IPA. The overall model fit for IPA-short version improved but was still below levels of acceptability (26-items: CFI = .79, TLI = .77, SRMR = .09;21-items: CFI = .89, TLI = .88, SRMR = −.07).

Including the 5th domain, Work and education, internal reliability was confirmed by Cronbach’s alpha (.81; *n* = 234). Item-test correlations ranged from .57 to .83. Sibley [15] found Cronbach’s alpha equal to .90 and item-total correlations .52–.77. Item-rest correlations were low (< 0.50) for two items (7c and 8).

CFA for the 5th domain, Work and education, gave close to acceptable fit indices: CFI .929, TLI .882 and SRMR .049. A high error covariance was found between items 7a “chances to get a job” and 7f “chances to find another job or employer” (MI 24.62, EPC.33). Therefore, item 7a was excluded from the model. The 5-item version of the Work and education domain showed lower but acceptable domain reliability (Cronbach alpha 0.77) and excellent model fit (CFI .998, TLI .996 and SRMR .024).

Factor analysis (PCF) with the complete IPA (adapted version, 26 items) revealed a 5-factor model in which all work-related items form a separate domain (factor loadings .46–.88). Contact with colleagues loaded high on SOC as well. Education loaded on AO. However, CFA showed a poor model fit (CFI .875, TLI .859, SRMR .073) (the results are available upon request from the first author).

#### Concluding

Validation of the original IPA domains for social-support clients showed a need to delete 6 items. The resulting 26-item IPA revealed a more or less similar classification of the items into the domains but lacks sufficient model fit. In the next section we proceed by adapting the questionnaire even further.

### Towards a participation model for the social-support population: IPA-MO^4^

#### 5 domain IPA-MO model: Autonomy indoors, family role, autonomy outdoors, social life & relations, and financial autonomy

For the construction of an adequate model for social-support clients, called IPA-MO, we proceeded with the short version IPA, including the three new items on financial issues and excluding the final item for reasons previously explained (see also Appendix). Principal component factoring with oblique rotation was used to determine the factor structure. For the total research population, a four-factor structure was found in which the IPA domains (AI, FR, SOC and AO) are visible with slight modifications: 1b (mobility indoors) moves from AI to FR, 5g (supporting other people) from SOC to AO. The financial items form a new domain that we call Financial Autonomy (FIN). FR and AO load on the same factor. The results are presented in Table 7.

**Table 7:**
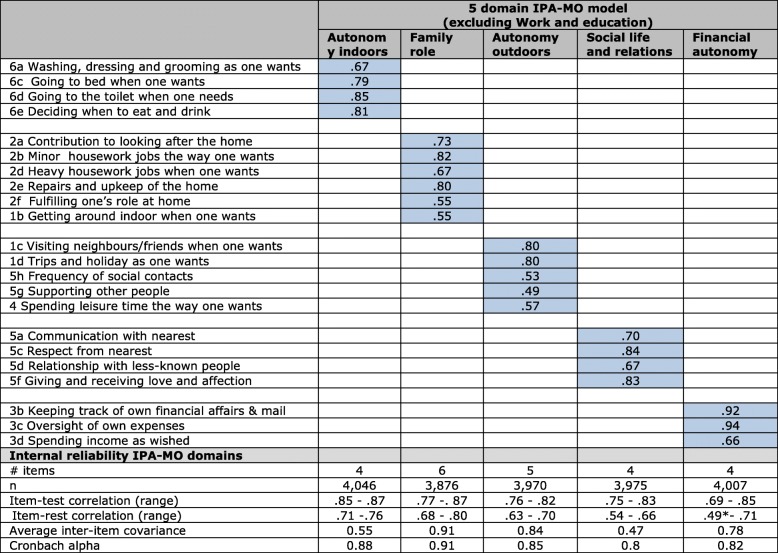
Rotated factor loadings IPA-MO model for social-support clients (principal component factoring, oblique rotation, *n* = 3301)

Source: IPA-MO database, 2014–2016

^a^one item (3a) below .50

All domains showed good reliability with Cronbach’s alpha’s ranging from .80 and .93. High inter-item covariances are observed in domains AO, FR and FIN. In the new domain on Financial autonomy, item 3a has a low correlation (.49) with the other items in this domain. Excluding this item gives a slightly higher alpha but very high inter-item covariance (.97) as well.

**Fig. 1:**
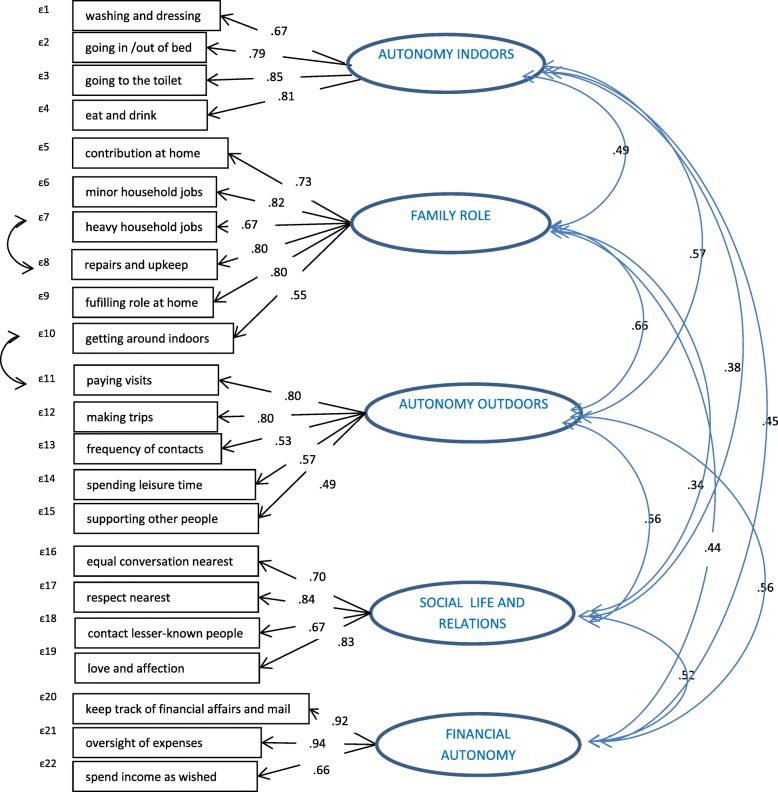
The IPA-MO model – perceived participation of social-support clients

CFA showed best model fit for a 5-domain model (AI, FR, AO, FIN, SOC) leaving out item 3a from the FIN domain (CFI .920, TLI .907, SRMR .053). High covariance errors between items still hampered model fit. Adding a path between the measurement errors of two pairs of items (1b-1c, 2d-2e) improves model fit to acceptable levels (CFI .936, TLI .925, SRMR .051). The path diagram of the model is represented in Fig. 1.

#### 6 domain IPA-MO model: adding work and education

Including the Work and education domain in the IPA-MO model, factor analysis (PCF) reveals a six-factor model in which each domain forms a separate factor. Item 7c ‘Contact with colleagues’ best fits in the SOC domain, leaving the Work and education domain with four items (7b, 7d, 7e, 8). Domain reliability is still acceptable (Cronbach’s alpha .78, item-test correlation .80–.90, item-rest correlation .37–.67, average inter-item covariance .65). Item 5 g ‘supporting others’ moved from AO to SOC, as in the original IPA. Domain composition and factor loadings are shown in Table 8. Model fit was on the verge of acceptability (CFI .906, TLI .892, SRMR .072). Including an additional path to correct for the highest error covariance (3b-3c) improved goodness of fit to acceptable values: CFI .915, TLI .903, SRMR .067.

**Table 8:** Domain composition and rotated factor loadings (PCF, Promax) for IPA-MO including work (*n* = 237)

Factor/domain	Items (rotated loadings)
Autonomy indoors	6a(.76) 6c (.67) 6d (.90) 6e (.72)
Family role	2a(.67) 2b (.55) 2d (.78) 2e (.74) 2f (.68) 1b (.17)
Social life and relations	5a(.80) 5c (.82) 5d (.58) 5f (.67) 5 g (.44) 7e(.49)
Autonomy outdoors	1c (.54) 1d (.74) 5 h (.53) 4 (.60)
Work and education	7b (.74) 7d (.94) 7e (.77) 8 (.36)
Financial autonomy	3b (.89) 3c (.91) 3d (.56)

Source: IPA-MO database, 2014–2016

### Model fit for subgroups

Using CFA, the 5-domain IPA-MO model was tested on goodness of fit for each one of the disability-based and age groups. Acceptable fit was confirmed for all of them. Differences between groups are small, only the group with severe physical disabilities clearly has lower fit indices than the others (Table 9).

**Table 9:** Model fit of IPA-MO model (5 domains)

Measure	Mild physical	Severe physical	Mild intellectual	Mental	Income support	18–35	36–50	51–66	67–80	81+
n=	916	652	649	550	627	483	733	902	625	651
CFI	.939	.914	.939	.927	.925	.935	.928	.923	.930	.933
TLI	.928	.899	.929	.915	.912	.924	.916	.910	.918	.921
SRMR	.053	.064	.049	.055	.057	.049	.059	.060	.060	.054

Source: IPA-MO database, 2014–2016

Exploratory factor analysis (PCF) revealed differences between groups in factor structure. For most groups, a 4-factor model was found with domains AO and FR located on the same factor. The items 5 h ‘frequency of social contacts’ and 5 g ‘supporting others’ often showed highest loadings on ‘SOC’. However, adapting the model structure accordingly did not improve model fit.

For the 81+ and the ‘mild physical disabilities’ group, all domains loaded on separate factors in a 5-factor model. Items 5 h and 5 g had significant loadings on AO only.

Table 10 summarises weighted scores for the IPA-MO participation domains for the total research population as well as individual client groups. All groups show highest scores on their autonomy at home (AI). The average score (3.88) indicates that people living with a disability generally manage well at home with the support they get. Lowest average scores are reported on the domains of Work and education (2.88), Autonomy outdoors (3.04) and Financial autonomy (3.06), indicating that interaction with society is more complicated. The average score for the possibility to live as one wants is just above ‘fair’ (3.12). The instrument reveals differences between subgroups. People with severe physical disabilities suffer most deficiencies in their autonomy and participation. Autonomy indoors, Autonomy outdoors and Family role diminish with age, while scores for Social life and relations, as well as living the way one wants, decline till the age group of 51–66 years and then go up again.

**Table 10:** Results of the IPA-MO questionnaire for the research population (*n* = 4120)

Participation domain^a^	Client group										
	All	Mild physical impairment	Severe physical	Mild cognitive	Mental impairment	Long-term income support	18–35 yrs	36–50 yrs	51–66 yrs	67–80 yrs	81+ yrs
Autonomy Indoors (AI)											
n =	4023	1162	784	743	628	706	545	829	1031	751	795
Weighted score	3.88	3.82	3.51	4.11	3.97	4.07	4.11	4.01	3.94	3.68	3.70
St dev	.79	.70	.87	.74	.73	.76	.77	.76	.76	.80	.77
% missing	2.4%	2.8%	3.9%	1.9%	0.5%	1.0%	1.3%	1.2%	1.6%	10.4%	4.7%
Family role (FR)											
n =	3723	1028	737	701	591	666	516	782	969	688	706
Weighted score	3.31	2.98	2.75	3.76	3.60	3.67	3.83	3.66	3.40	2.84	2.84
St. dev	1.00	.91	.98	. 90	.89	.90	.90	.89	.94	.95	.95
% missing	9.6%	14.6%	9.7%	7.4%	6.3%	6.6%	6.5%	6.8%	7.5%	10.4%	15.4%
Autonomy outdoors (AO)											
n =	3786	1061	727	721	596	681	528	805	986	696	710
Weighted score	3.04	2.82	2.52	3.55	3.21	3.25	3.58	3.29	3.09	2.73	2.59
St. dev	.99	.88	.95	.93	.96	.93	.95	.92	.96	.93	.92
% missing	8.1%	11.8%	10.9%	4.8%	5.6%	4.5%	4.4%	4.1%	5.9%	9.4%	14.9%
Social life & relations (SOC)											
n =	3917	1129	769	721	609	689	528	803	1020	727	772
Weighted score	3.56	3.74	3.36	3.56	3.37	3.66	3.62	3.53	3.48	3.55	3.67
St. dev	.77	.66	.76	.79	.85	.79	.82	.77	.83	.74	.67
% missing	4.9%	6.2%	5.8%	4.8%	3.5%	3.4%	4.4%	4.3%	2.7%	5.3%	7.4%
Financial autonomy (FIN)											
n =	3972	1158	785	727	607	695	530	812	1016	743	802
Weighted score	3.06	3.39	2.57	2.86	3.05	3.28	3.03	3.02	3.10	3.05	3.08
St. dev	1.09	.95	1.11	1.17	1.06	.95	1.16	1.06	1.05	1.13	1.06
% missing	3.6%	3.7%	3.8%	4.0%	3.8%	2.5%	4.0%	3.2%	3.1%	3.3%	3.8%
Work & education (WORK - based on items 7d 7f 7g 8)											
n =	268	10	17	70	84	87	77	111	70	–	–
Weighted score	2.88	**–**	**–**	3.13	2.85	2.82	3.10	2.88	2.61	–	**–**
St. dev	0.93	–	–	0.90	0.97	0.94	0.90	0.89	0.99	–	–
% missing	93.5%	99.2%	97.9%	90.8%	86.7%	87.7%	86.1%	86.8%	93.3%	100%	100%
Possibility to live the way one wants											
n =	3976	1143	779	739	617	698	541	821	1022	735	787
Mean score	3.12	3.17	2.89	3.54	3.05	2.94	3.37	3.09	3.03	3.06	3.18
St. dev	1.09	1.03	1.09	1.01	1.14	1.09	1.11	1.10	1.11	1.07	1.02
% missing	3.5%	5.0%	4.5%	2.4%	2.2%	2.1%	2.0%	2.2%	2.5%	4.3%	5.6%

Source: IPA-MO database, 2014–2016

Domain scores were calculated for respondents only if all items had been answered

^a^Scores: 1 = very poor 2 = poor 3 = fair 4 = good 5 = very good

## Discussion

The present study examined the reliability and validity of the IPA instrument adapted for social-support clients, called IPA-MO. We used data from large cohorts of social support populations (*n* = 4120) from various parts of the Netherlands. The research sample comprised the full range of client types that municipalities have to support: elderly people with mild and severe physical disabilities, people with mild cognitive and mental conditions, and inhabitants dependent on income support. In order to create more homogeneous groups, both impairment-based and age-based groups were distinguished and analysed in this study.

We found that IPA-MO is a reliable and valid instrument for this population and for each one of the homogeneous client groups. Five participation domains are identified: Autonomy indoors, Family role, Autonomy outdoors, Social life and relations (as in IPA), and Financial autonomy as a new domain. CFA showed acceptable fit of this model for the whole research population and for all subgroups separately, though fit was marginally acceptable for people with severe physical disabilities.

Six items of the original IPA questionnaire were eliminated in the IPA-MO model since they showed high correlation with other items. Whereas nearly identical items may be useful when the questionnaire is used on an individual basis – as in rehabilitation practice –, in our surveys these items hardly added any information and hurt validity of the instrument by causing high error covariances. The shortened domains showed good reliability, high conceptual similarity with the original domains and resulted in acceptable fit for the overall model. Elimination of highly correlated items is often applied when screening instruments from medical practice are used for research purposes, also for efficiency purposes [33].

All participation domains of the original IPA model proved valid for social-support clients, though minor changes occur. One of the most salient changes is that the item on reciprocity (‘supporting other people’) is related to Autonomy outdoors instead of to Social life and relations. In studies among elderly people, Haak [34] and Sixsmith [35] found that ‘doing things for others’ is an important basis for participation of aged people and strengthens their personal identity. Elderly people form a minority in our research population so this may also be true for younger persons that do not participate in the labour process. The fact that supporting other people did not contribute to Autonomy outdoors for participants with work, further supports this assumption. Since reciprocity is an important feature of a participation society, its potential to improve participation deserves further research.

Financial autonomy is a new participation domain as a result of two new items added to the original single item on this subject. The new items are derived from an institutional screening instrument. Whereas this domain is statistically reliable and valid, its items are few and conceptually almost identical (extremely high inter-item covariance). Further research is recommended to investigate if clients’ perception of financial autonomy is sufficiently covered.

Validation of the participation domain Work and education was based on a small group of participants due to high non-response. Whereas good domain reliability was found, validity needs further testing. In our sample, education proved to correlate weakly with work items, and contact with colleagues loaded onto Social life and relations.

Based on the analysis in this study, both disability-related groups and age groups are statistically valid options to identify homogeneous subgroups within the social-support population. Disability-related subgroups have higher discriminatory power as they reveal larger differences, both in terms of model fit and scores on participation domains and perceived well-being.

The domains in our model are all interrelated. Strongest interrelation is found for Autonomy outdoors, Family role and Autonomy indoors. An IPA study among rehabilitation clients in Iran showed similar findings, to the extent that Autonomy outdoors, Family role and Autonomy indoors were combined into one domain [19]. The new domain Financial autonomy shows the highest interrelation with Autonomy outdoors and Social life and relations. For our study population, Autonomy outdoors and Family role load onto the same factor for most groups, yet model fit is better when they are treated as separate domains.

By its very nature, Autonomy outdoors – meeting and supporting people, spending leisure time, paying visits, making trips – represents the way people participate in society. The near-symbiosis with Family role suggests that for social-support clients, Autonomy outdoors is closely related to home-based activities. As most of them do not work and many live alone, their connection with society seems weak. This is probably (part of) the reason why these people receive social support. A relevant question for the aspired transformation towards a participation society is whether social support can reinforce connections with and improve participation in society. The IPA-MO instrument can be helpful to monitor this development.

Reliable outcome measurement is a key element for outcome-oriented policies (Brickley et al. [8]). Most local governments in the Netherlands are reorganising their social support interventions so as to achieve more efficiency and avoid budget deficits: e.g. family members are expected to contribute more to supporting their relatives and general facilities are being created to replace individual support. Whereas this decreases expenditure, the social impact of these policy measures remains largely unknown. The IPA-MO instrument provides an option for local governments to measure the social outcomes of their policies in a reliable way. For this purpose, they can conduct periodic surveys among a representative sample of inhabitants living with disabilities. As the IPA-MO amounts to an overall score representing perceived autonomy and participation and weights for domain scores can be established (further research), the instrument can be used for social cost-benefit analyses [36] which allows insight into the overall impact of social policies.

Having a valid instrument at their disposal to measure outcomes, also offers municipalities possibilities to contract integrated care or care-service packages, and replace the fee-for-service with value-based reimbursement systems. This will shift the incentive for providers from production to more meaningful results and it is expected to encourage innovation and reduce the administrative burden in social support.

Finally, outcome measurement by IPA-MO puts clients’ perspectives at the centre of policy making. Clients are numerous and dispersed and, in spite of the best intentions, hard to involve widely in policy processes. Taking their perceptions of autonomy and participation as the acid test for the effectiveness of social support policies, may prove a serious game-changer in politics.

## Conclusions

This study demonstrates that the IPA-MO is a valid and reliable instrument to assess outcomes of social support in terms of self-reliance and participation among heterogeneous populations, including people that live with physical, intellectual or mental disabilities as well as people dependent on income support. The instrument was built on the existing and validated IPA instrument for rehabilitation clients and adapted for the purpose of impact measurement of social support policies among the target population of local governments. In comparison with the original IPA, several items were eliminated and a new domain Financial autonomy was included. Further research is needed on face validity and responsiveness of this new domain. The IPA-MO instrument allows for the distinction of subgroups based on age or nature of the disability, which provides local policy makers with more specific information on autonomy and participation so as to target policies to the potential and needs of different groups.

## Appendix

**Table 11:** The IPA-MO questionnaire used for social-support clients

Structure/items questionnaire	IPA Original	IPA-MO Social support^a^
Mobility		
1a. My chances of getting around in my house ***where*** I want are	+	correlated
1b. My chances of getting around in my house ***when*** I want are	+	+
1c. My chances of visiting neighbours, friends and relatives ***when*** I want are	+	+
1d. My chances of going on the sort of trips and holidays I want are	+	+
Tasks and activities in and around home		
2a. My chances of contributing to looking after my home ***the way*** I want to are	+	+
2b. My chances of getting light tasks done around the house (e.g. making tea or coffee) either by myself or others, ***the way*** I want, are	+	+
2c. My chances of getting heavy tasks done around the house (e.g. cleaning), either by myself or by others, ***the way*** I want are	+	correlated
2d. My chances of getting heavy tasks done around the house (e.g. cleaning), either by myself or by others, ***when*** I want them done are	+	+
2e. My chances of getting minor repairs and maintenance work done in my house and garden, either by myself or others, ***the way*** I want them done are	+	+
2f. My chances of fulfilling my role at home (e.g. as partner, parent or boss in my own home) ***as*** I would like to are	+	+
Financial issues		
3a. My chances of paying with my income what is ***really necessary***, are		added
3b. My chances of ***keeping track*** of my financial affairs and mail are		added
3c. My chances to maintain ***oversight of my expenses*** are		added
3d. My chances to choose ***how*** I spend my money are	+	+
Leisure time		
4. My chances of using leisure time *the way* I want to are	+	+
Social contacts and relationships		
5a. My chances of talking to people close to me on equal terms are	+	+
5b. The quality of my relationships with people close to me is	+	correlated
5c. The respect I receive from people who are close to me is	+	+
5d. The quality of my relationships with people I do not know very well is	+	+
5e. The respect I receive from people I do not know very well is	+	correlated
5f. My chances to give and receive love and affection are	+	+
5g. My chances to support people who need me are	+	+
5h. My chances of seeing people as often as I want are	+	+
Self care		
6a. My chances of getting washed and dressed ***the way*** I want are	+	+
6b. My chances of getting washed and dressed ***when*** I want are	+	correlated
6c. My chances of getting up and going to bed ***when*** I want are	+	+
6d. My chances of going to the toilet ***when*** I wish and need to are	+	+
6e. My chances of eating and drinking ***when*** I want to are	+	+
Paid or voluntary work		
(7a. My chances of getting a paid or voluntary job ***I would like to do*** are)	(+)	correlated
7b. My chances of doing my work ***the way*** I want are	+	+
7c. My contacts with colleagues at my work are	+	+
7d. My chances of achieving or keeping the position ***I want*** in my work are	+	+
7e. My chances of getting different work or another employer are	+	+
Education and training		
8. My chances of continuing or starting the education or training I want are	+	+
Overall		
9. My chances of living life the way I want to are	+	Outside the analysis
Total items	32	26

Added: these items are added to the original IPA questionnaire

Outside the analysis: as a concluding item, it is meant and phrased as an overall appreciation rather than a predictor of perceived participation

^a^Correlated: In the questionnaire, but this item correlates with another item and therefore it is not part of a domain

## Abbreviations

ADHD: Attention deficit hyperactivity disorder
AI: Autonomy indoors
ALS: Amyotrophic lateral sclerosis
AO: Autonomy outdoors
CFA: Confirmatory Factor Analysis
CFI: Comparative Fit Index
EFA: Exploratory Factor Analysis
FIN: Financial autonomy
FR: Family role
ICF: International Classification of Functioning, Disability and Health
IPA: Impact on Participation and Autonomy questionnaire
IPA-MO: Impact on Participation and Autonomy questionnaire of Social Support
PCF: Principal Component Factoring
Sem: Structural equation modelling
SOC: Social life and relations
SRMR: Standardised roots mean squared residual
TLI: Tucker Lewis Index
WMO: Social Support Act (Wet Maatschappelijke Ondersteuning)
WORK: Work and education

## Footnotes

1 Following the instruction manual, sections of the questionnaire are invalid when less than 75% of the items have been answered

2 Up to the age of 95 years old, the majority of Dutch inhabitants live independently. Under the Social Support Act (Wmo), local governments have the responsibility to assist them with technical aids and ambulant support if needed. Given the ageing Dutch population and the fact that government policies have restricted entrance to intramural care, the number of elderly Wmo clients is likely to increase [37]

3 E.g. 6a is “Getting washed and dressed the way I want” and 6b is “getting washed and dressed when I want”

4 MO refers to Maatschappelijke Ondersteuning (Social Support)
